# Conference of the Slaughter-house Directors and Representatives of the Cities with Public Cattle-yards in Berlin

**Published:** 1896-09

**Authors:** 


CONFERENCE OF THE SLAUGHTER-HOUSE DIRECTORS AND REPRESENTATIVES OF THE CITIES WITH PUBLIC CATTLE-YARDS IN BERLIN. In pursuance of a proposition made by Mayor Zelle, of Berlin, last April, that representatives of other cities which possess stock-yards, abattoirs, etc., should be invited to a conference in Berlin to discuss the questions relative to cattle-insurance, the regulation against the spread of epidemics, the fixation of the remuneration for condemned animals, and the general interests of the cattle-yards and slaughter-houses, representatives from forty-seven cities in Germany, as well as from Vienna and Prague, assembled in Berlin, and held a conference which lasted from May 12th to 15th. The programme was quite extensive. The following resolutions were adopted:

1. The cattle-insurance question. A resolution was passed requesting the Imperial Chancellor and the several provincial governments to make, by legal enactment as soon as possible, the insurance of cattle compulsory without financial burden to the communities; and, further, if a general law of this nature could not be passed, that the authority to pass similar laws be given to those provinces which have already introduced the compulsory inspection of meat.

2. The technical utilization of condemned animals. *Resolved:* It is highly desirable that in all cattle-yards and in places connected with the same arrangements as complete as possible be made by means of which all condemned meat unfit for food may be, with full exclusion of the possibility of its being used as food, utilized in the interests of the owner; and by means of which slaughter animals, which in their fresh, raw condition are to be regarded as injurious to health, may be, under official oversight, treated and utilized in such a manner that their use as food will no longer prove injurious to health.

3. The introduction of a general law in regard to the inspection of meat. *Resolved:* The assembly of delegates recommends that the attention of the Imperial Chancellor and the ministry be called to the immediate necessity of introducing a general law for the whole of Germany regarding meat-inspection; this law to be framed after that of the Kingdom of Saxony and the South German States.

4. The question of the prevention of cattle epidemics.

*Resolution* I. The assembly recommends the following amendments to the imperial epidemic law of June 27, 1895: 1. In cattle-yards which are under the control of the regular veterinary police only those cattle and hogs are to be regarded as suspects of infection which have been placed in the same stable with a sick animal or one suspected of the epidemic. In this case the stable is to be quarantined. Quarantine of the whole pen can only take place when the presence of the epidemic simultaneously in several stables is officially confirmed. 2. In larger and isolated localities where there is a cattle-yard, if an outbreak of some epidemic takes place, but the cattle-yard is to be regarded free from the epidemic, then the law prohibiting the holding of a cattle-market is not to be extended to the cattle-yard. On the other hand, if the epidemic appear only in the cattle-yard, and the same must be quarantined, then the law prohibiting the holding of a cattle-market is to be applied only to the cattle-yard. 3. The epidemic is to be considered over and the quarantine and preventive regulations are to be suspended in the cattle-yards under veterinary control as soon as the cattle and swine in quarantine have either been killed or removed to some isolated yard for infected cattle and the regular disinfection has taken place. II. Efforts are to be made to bring the following points and considerations to the attention of the Bundesrath: 1. Those places in which regular and often large sales of cattle are held must be so constructed that they can be easily disinfected. 2. In case the foot-and-mouth epidemic is widely spread in the several States the general government should appoint an epidemic commissioner. 3. A uniform method of examination in the case of animals with foot-and-mouth disease is to be aimed at, as well as in the form and contents of the symptoms of health, so that the regulations of one State shall have full validity in all.—*Thierärzt. Centralblatt*, May 1, and June 1, 1896.

